# Mesenteric Diatrizoate Pooling Mimicking an Anastomotic Leak in Multi‐Organ Dysfunction

**DOI:** 10.1002/ccr3.73100

**Published:** 2026-07-05

**Authors:** Ryusuke Katsuki, Masanobu Tsuda, Takayuki Irahara, Takayuki Kai, Dai Oishi, Tsuguaki Terashima, Eizo Watanabe

**Affiliations:** ^1^ Advanced Critical Care Center Aichi Medical University Nagakute Aichi Japan

**Keywords:** acute medicine, critical care medicine, emergency medicine, radiology and imaging, surgery

## Abstract

In patients with multi‐organ dysfunction following severe physiological insult, such as prolonged cardiac arrest, enteral diatrizoate can be transmurally absorbed and sequestered within a contused mesentery. This retention mimics an active anastomotic leak on CT; clinical stability and the absence of fluid collections are essential to avoiding unnecessary re‐intervention.

## 1 Case Description

A 68‐year‐old man sustained blunt abdominal trauma, necessitating resuscitative thoracotomy with aortic cross‐clamping for 28 min and laparotomy for mesenteric and small‐bowel injuries. The initial damage control surgery involved stapled resection of the injured small bowel with open abdomen management. At second‐look laparotomy 24 h later, the entire ileum was found necrotic secondary to mesenteric vascular compromise; it was resected, and intestinal continuity was restored between the remaining small bowel and the cecum via a functional end‐to‐end anastomosis using a single‐layer Gambee technique. No stoma was created. Intraoperative cardiac arrest lasted a total of 18 min, leading to profound systemic hypoperfusion. Postoperatively, he developed severe hepatic dysfunction (total bilirubin 17.5 mg/dL) and acute kidney injury (creatinine 3.92 mg/dL). Parenteral nutrition was commenced on postoperative day (POD) 4 and transitioned to enteral nutrition on POD 14.

**FIGURE 1:**
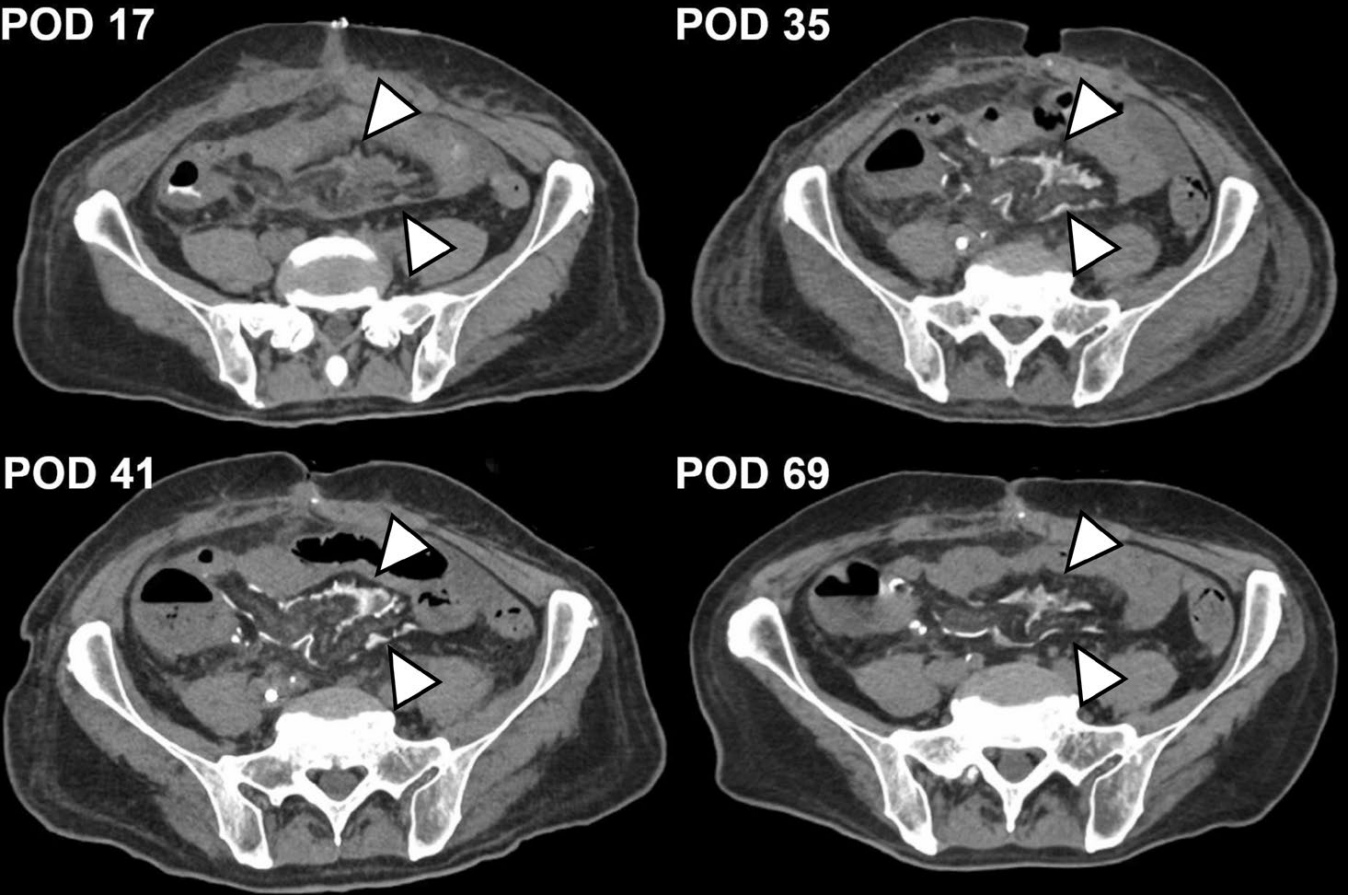
Sequential non‐contrast CT images. On POD 17, punctate high‐attenuation appeared within the contused mesentery (white arrows) after enteral diatrizoate administration. The attenuation intensified by POD 35 and 41, eventually fading by POD 69 as the patient's organ function improved. Note the circular high‐attenuation in the right common iliac vein on POD 35 and 41 represents an indwelling hemodiafiltration catheter.

On POD 17, enteral diatrizoate (Gastrografin, Bayer) [1] was administered via a nasoduodenal tube to confirm tube position during advancement to the jejunum. Non‐contrast CT showed punctate‐to‐curvilinear high‐attenuation within the contused mesentery (Figure 1, arrows), mimicking an extraluminal leak from the ileocecal anastomosis. However, there was no extravasation at the anastomosis, no pneumoperitoneum, no opacification of the urinary tract, and no free fluid or collection in the pelvis or abdominal cavity. Despite the concerning radiology, the patient remained clinically stable, tolerated continuous enteral feeding, and had negative peritoneal cultures.

The mesenteric high‐attenuation intensified at POD 35 and 41 before gradually fading by POD 69, mirroring the recovery of hepatic and renal functions (total bilirubin/creatinine improved to 3.2/0.68 mg/dL by POD 69). While Gastrografin is primarily eliminated via the gut, approximately 3% is normally absorbed and renally excreted. In ischemic or damaged bowel, transmural absorption can increase [2]. In this case, we hypothesize that the absorbed contrast extravasated into the contused mesentery and was sequestered due to impaired local lymphatic drainage and delayed systemic clearance associated with multi‐organ failure. Clinicians should prioritize the overall clinical course to avoid misinterpreting isolated mesenteric high‐attenuation as an active leak in complex post‐traumatic settings [3].

## Author Contributions

**Ryusuke Katsuki:** conceptualization, data curation, writing – original draft. **Masanobu Tsuda:** conceptualization, data curation, writing – review and editing. **Takayuki Irahara:** conceptualization, data curation, writing – review and editing. **Takayuki Kai:** conceptualization, data curation, writing – review and editing. **Dai Oishi:** conceptualization, data curation. **Tsuguaki Terashima:** conceptualization, data curation. **Eizo Watanabe:** conceptualization, supervision, writing – review and editing.

## Funding

The authors have nothing to report.

## Consent

Written informed consent was obtained from the patient for publication of this case report and accompanying images.

## Conflicts of Interest

The authors declare no conflicts of interest.

## Data Availability

The data that support the findings of this study are available from the corresponding author upon reasonable request.
